# An exploration of inter-organisational partnership assessment tools in the context of Australian Aboriginal-mainstream partnerships: a scoping review of the literature

**DOI:** 10.1186/s12889-015-1537-4

**Published:** 2015-04-23

**Authors:** Christina Tsou, Emma Haynes, Wayne D Warner, Gordon Gray, Sandra C Thompson

**Affiliations:** Western Australian Centre for Rural Health (WACRH), University of Western Australia; Inner East Primary Care Partnership, 6 Lakeside Drive, Burwood East, VIC 3151 Australia; Western Australian Centre for Rural Health (WACRH), University of Western Australia, M706, 35 Stirling Highway, Stirling, 6009 WA Australia; Western Australian Centre for Rural Health (WACRH), University of Western Australia, 167 Fitzgerald St, Geraldton, 6530 WA Australia; Midwest Aboriginal Organisations Alliance (MAOA), Eastward Rd, Utakarra, WA 6530 Australia

**Keywords:** Partnership, Assessment, Tools, Evaluation, Applicability, Aboriginal-mainstream partnership, Indigenous

## Abstract

**Background:**

The need for better partnerships between Aboriginal organisations and mainstream agencies demands attention on process and relational elements of these partnerships, and improving partnership functioning through transformative or iterative evaluation procedures. This paper presents the findings of a literature review which examines the usefulness of existing partnership tools to the Australian Aboriginal-mainstream partnership (AMP) context.

**Methods:**

Three sets of best practice principles for successful AMP were selected based on authors’ knowledge and experience. Items in each set of principles were separated into process and relational elements and used to guide the analysis of partnership assessment tools. The review and analysis of partnership assessment tools were conducted in three distinct but related parts. Part 1- identify and select reviews of partnership tools; part 2 – identify and select partnership self-assessment tool; part 3 – analysis of selected tools using AMP principles.

**Results:**

The focus on relational and process elements in the partnership tools reviewed is consistent with the focus of Australian AMP principles by reconciliation advocates; however, historical context, lived experience, cultural context and approaches of Australian Aboriginal people represent key deficiencies in the tools reviewed. The overall assessment indicated that the New York Partnership Self-Assessment Tool and the VicHealth Partnership Analysis Tools reflect the greatest number of AMP principles followed by the Nuffield Partnership Assessment Tool. The New York PSAT has the strongest alignment with the relational elements while VicHealth and Nuffield tools showed greatest alignment with the process elements in the chosen AMP principles.

**Conclusions:**

Partnership tools offer opportunities for providing evidence based support to partnership development. The multiplicity of tools in existence and the reported uniqueness of each partnership, mean the development of a generic partnership analysis for AMP may not be a viable option for future effort.

## Background

Australia, along with other high income countries with colonial pasts, has struggled to minimise the disparities between its Aboriginal and non-Aboriginal populations, where the gap in life expectancy is variously reported as being between 11 and 18 years [1]. Partnerships between Aboriginal and mainstream organisations are seen as fundamental for improving Aboriginal health outcomes [2-7].

Aboriginal community controlled organisations, led by Aboriginal boards, provide culturally appropriate services as well as reflecting the aspirations Aboriginal people have for self-determination [8]. Many of these corporations and services are now significant enterprises [9,10] and have important roles in supporting mainstream delivery of effective services. For example, many Aboriginal Community Controlled Health Services (ACCHS) in addition to providing programs and services directly to their local Aboriginal community often support mainstream organisations to access community members by allowing them utilise their infrastructure [11-13], and participate in partnerships to deliver services or conduct research [14-21]. Baeza and Lewis also point out the role that ACCHSs have played in educating mainstream services on providing appropriate care for and working with Aboriginal people [22].

Successful Aboriginal-mainstream partnerships (AMP) offer multiple opportunities for improving health outcomes [2,3,5] and community development, particularly in rural and remote Australia [23]. However, with poor Indigenous health outcomes/indices widely attributed to colonisation and its ongoing expressions [24-26], building such partnerships can be challenging and complex particularly as they continue to be affected by Australia’s historical and current context of race and political relations [5].

Partnerships are also affected by disparate ways of working; power dynamics and funding timeline pressures to deliver outputs that often overwhelm the necessary development of trust and relationships [17,27,28]. ACCHS face particular governance challenges balancing community obligations with financing and reporting accountabilities that impact on their ability to maintain partnerships [29].

Partnerships are particularly undermined and collaborations weakened through lack of clarity about respective roles resulting in confusion about the partnership’s purpose, its objectives and how to measure its success [5,30]. The ability to assess the success of Aboriginal-mainstream partnerships is therefore critical in both communicating the value of the partnership internally and externally and to ensure continued growth and strengthening of the partnership [27].

### Objective of the review

Approaches to assessing and improving Aboriginal-mainstream partnerships, and the value of using appropriate tools, remain open to debate. This scoping review [31] explores existing partnership assessment tools for their potential value in measuring the success of AMP and supporting partnership development. As partnership assessment is intrinsically linked to developing and strengthening partnerships, the review focuses on self-assessment tools, not external evaluation processes.

While an independent facilitator may usefully be involved in supporting partnership self-assessment, the tools reviewed here demand a degree of participation by the partners themselves. As suggested by Taylor and Thompson, the focus of partnerships needs to be on developing genuine, trusting relationships that are tangibly linked to the Aboriginal community. ‘Failure to invest in this relational process and push forward with ‘business as usual’ can ultimately have negative ramifications on client outcomes’ [5]. This review therefore focuses on partnership assessment tools that emphasize the importance of understanding successful partnerships, recognising that both process and relational factors are important in successful partnerships [5].

## Methods

### Review and analysis of partnership assessment tools

The review was conducted in three distinct but related parts, described in detail below.

#### Part 1: identifying and selecting reviews of partnership tools

A search was conducted in October 2012 and updated in October 2014 using Science Direct, Proquest, Sage, PubMed, Informit, Wiley Online (Journal), and Google Scholar and the key phrases: partnership assessment tools review, partnership tool applicability, partnership tool application, and Aboriginal-mainstream partnership. Additional searching was undertaken using the Google search engine to identify relevant additional literature not captured in the previous search.

Articles were excluded from further consideration if they did not have specific reference to partnership evaluation procedures or were reviews of partnership tool analysis without a clear documentation of methodology. The remaining articles were reviewed to identify potential partnership self-assessment tools (see Part 2 below), and learnings regarding implementing tools.

#### Part 2: identifying and selecting partnership self-assessment tools

Partnership assessment tools identified from the articles identified in Part 1 were downloaded for detailed review. The inclusion criteria for partnership tools were set as follows:

1. elements included in tools were based on a literature review or systematic examination of multiple practitioner experiences of a successful partnership, or were included in at least two of the tool review articles found in the first part of this analysis;
2. tools were designed to facilitate self-assessment and contain checklist or Likert scale-like components as opposed to a conceptual framework, open-ended questions or activity instructions to support partnership design or the partnering process;
3. tools were openly accessible from the internet.

Partnership tools designed to foster partnership within a single organisation were excluded as our interest was in AMP as inter-organisational partnerships.

#### Part 3: analysis of selected tools using AMP principles

**Figure 1:**
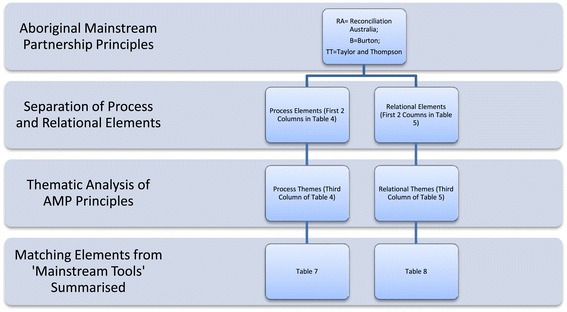
Thematic and gap analysis including quick reference to results tables.

Process and relational elements within the items in the identified AMP principles were identified. Similarly, process and relational elements contained in the partnership self-assessment tools were also identified. Process and relational AMP principles were then used as the basis to assess the usefulness of partnership assessment tools to AMP. This process identified elements in the partnership assessment tools appropriate to the AMP context as well as gaps or deficiencies in assessing adherence of partnerships to identified AMP principles. Figure 1 summarises this analysis, including references to the tables in which results are documented.

## Results

### Findings from literature search

**Figure 2:**
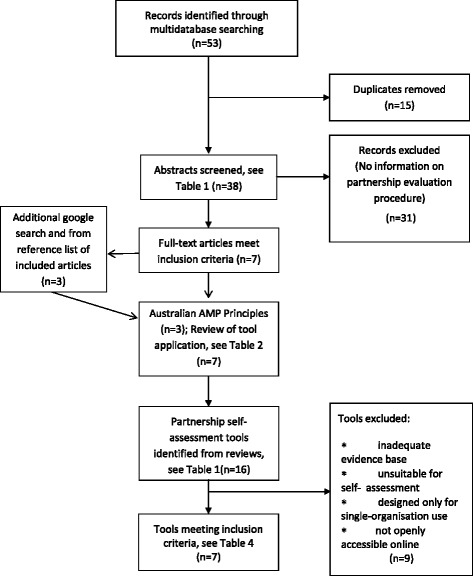
Stages of literature sourcing, screening and examination.

The output from database searches and processes of eliminating articles for detailed review and analysis are summarised in Figure 2. Three broad types of data sources were identified: AMP principles, review of partnership tools or documentation of experience in applying partnership tools in inter-organisational partnerships and partnership self-assessment tools.

Table 1 summarises articles included for abstract review and initial list of partnership tools reviewed against inclusion criteria.

**Table 1:** **Abstracts and partnership self-assessment tools reviewed**

**Reference**	**Aboriginal-mainstream partnership discussions**	**Partnership evaluation sourcebook**	**Partnership evaluation frameworks**	**Literature reviews on partnership assessment tools/roles of tools**	**Partnership building framework, guidelines or tools**	**Partnership self-assessment tools (containing bench marking check lists)**	**Nationality**
**Aboriginal and Torres Strait Islander Health: current status and recent initiatives** (Aboriginal and Torres Strait Islander Commission, 1993) [32]	v						AUS
**Successful Partnerships are the Key to Improving Aboriginal Health** (Bailey, 2012) [2]	v						AUS
**Opening Doors Through Partnerships** (Burton, 2012) [33]	v						AUS
**Achieving highly successful multiple agency collaborations in a cross-cultural environment: experiences and lessons from Dhimurru Aboriginal Corporation and partners** (Hoffman, 2012) [34]	v						AUS
**Closing the Gap report 2013: progress outcome for Aboriginal and Torres Strait Islander Peoples** (ACT Government, 2013) [35]	v						AUS
**Ten principles relevant to health research among Indigenous Australian population** (Jamieson, 2012) [36]	v						AUS
**Successful chronic disease care for Aboriginal Australian requires cultural competence** (Liaw, 2011) [19]	v						AUS
**Evidence-based policy making in Aboriginal and Torres Strait Islander Health** (Larkin, 2006) [37]	v						AUS
**Closing the (service) gap: exploring partnerships between Aboriginal and mainstream health services** (Taylor, 2011) [5]	v						AUS
**Partnership Working: A Consumer Guide to Resources** (Markwell, 2003) [38]		v					UK
**Sourcebook for Evaluating Global and Regional Partnership Programs: Indicative Principles and Standards** (World Bank, 2007) [39]		v				v	USA
**Using the Give-Get Grid to Understand Potential Expectations of Engagement in a Community-Academic Partnership** (Southerland, 2013) [40]			v				USA
**Operationalizing he RE-AIM framework to evaluate the impact of multi-sector partnerships** (Sweet, 2014) [41]			v				Canada
**The Development of an Evaluation Framework for Partnership Working** (Atkinson, 2005) [42]			v				UK
**Understanding Evaluation Influence Within Public Sector Partnership: a conceptual model** (Appleton-Dyer, 2012) [43]			v				USA
**Partnership Indicators. Measuring effectiveness of multi-sector approaches to service provision. BPD Water and Sanitation Cluster** (Caplan, 2002) [44]			v				UK
**A Partnership Model for Ethical Indigenous Research** (de Crespigny, 2004) [45]	v		v				AUS
**An evaluation of an Australian initiative designed to improve interdisciplinary collatoration in primary mental health care.** (Fletcher, 2014) [46]			v				AUS
**Integrating Assessment and Evaluation Into Partnership Initiatives: Lessons From the Community Partnerships for Older Adults** (Giunta, 2013) [47]			v				USA
**Partnerships between health and social services: developing a framework for evaluation** (Glendinning, 2002) [48]			v				UK
**Understanding Partnerships for Sustainable Development Analytically: the ladder of partnership activity as a methodological tool** (Glasbergen, 2011) [49]			v				DUTCH
**A model of output specifications for public-private partnership projects** (Javed, 2013) [50]			v				HK
**Partnership Evaluation** (Rieker, 2010) [51]			v				USA
**Making a Partnership Work: Outcomes Assessment of the Manufacturing Engineering Education Partnership** (de Ramirez, 1998 [52]			v				USA
**Improving partnership governance: using a network approach to evaluate partnerships in Victoria** (Pope, 2008) [53]			v				AUS
**Partnership synergy: a practical framework for studying and strengthening the collaborative advantage** (Lasker, 2001) [54]			v				USA
**Evaluation of partnership working within a community planning context** (Lamie, 2010) [55]			v				UK
**Partnership Literature Review and Framework** (Vance, 2004) [56]				v			USA
**The Evaluation of Partnership Working in the Delivery of Health and Social Care** (Ball, 2010) [57]				v			UK
**Acceptability of participatory social network analysis for problem-solving in Australian Aboriginal health service partnerships** (Fuller, 2012) [58]				v			AUS
**Evaluation community coalition characteristics and functioning: a summary of measurement tools** (Granner, 2004) [59]				v			USA
**Evaluating Partnership: The Role of Formal Assessment Tool** (Halliday, 2004) [60]				v			UK
**Perspectives on partnership: A literature review** (Horton, 2009) [61]				v			PE
**Partnership tools for health promotion: are they worth the effort?** (Joss, 2011) [62]				v			AUS
**A study tour of the UK, Canada, New Zealand on partnership management in primary health care focusing on governance, leadership, partnership evaluation and clinical governance across network partners** (Pietsch, 2006) [63]				v			AUS
**Current practice in the evaluation of cross-sector partnerships for sustainable development** (Serafin, 2008) [64]				v			UK
**How to create successful partnerships-a literature review** (Wildridge, 2004) [65]					v		UK
**Identifying value indicators and social capital in community health partnerships** (Hausman, 2005) [66]					v		USA
**Development and evaluation of a toolkit to assess partnership readiness for community-based participatory research** (Andrews, 2011) [67]						v	USA
**CGIAR Organisational Change Program Partnership Self-Assessment Inventory. Successful Collaborative Partnerships: Key Elements and a Self–Assessment Inventory** (Spink, 1999) [68]						v	USA
**Guidelines for Assessing Partnership Performance in Water and Sanitation. Assessing partnership performance: Understanding the drivers for success** (Caplan, 2007) [69]						v	UK
**International Food Policy Research Institute Guidelines for Public-private Partnerships for Agricultural Innovation** (Hartwich, 2007) [70]						v	USA
**One World Trust Toolkit for Accountability in Research Organisations** (Whitty, 2008) [71]						v	UK
**Partnership Self-Assessment Toolkit** (Frearson, 2002) [72]						v	UK
**A Fruitful Partnership – Effective Partnership Working** (Audit Commission for NHS, 1998) [73]						v	UK
**The New York Partnership Self-Assessment Tool** (Center for the Advancement of Collaborative Strategies in Health, 2002) [74]						v	USA
**EQUAL Guide for Development Partnerships** (European Commission, 2005) [75]						v	EU
**Partnership Building: Practical Tools to Help You Create, Strengthen, Assess and Manage Your Partnership or Alliance More Productively** (Gormley, 2007) [76]						v	USA
**Assessing Strategic Partnership: The Nuffield Partnership Assessment Tool** (Hardy, 2003) [77]						v	UK
**The Verona Benchmark Tool** (Markwell, 2003) [78]						v	UK
**Collaboration: What Makes It Work** (Mattessich, 2001) [79]						v	USA
**Partnerships and networks: an evaluation and development manual** (McCabe, 1997) [80]						v	UK
**The Partnering toolbook: An essential guide to cross-sector partnering** (Tennyson, 2011) [81]						v	UK
**The partnership analysis tool** (VicHealth, 2011) [82]						v	AUS

### Identification and selection of Aboriginal and mainstream partnership principles

To determine the suitability of existing partnership assessment tools required identifying best practice principles for satisfactory AMP functioning in Australian context. We selected from three key sources [5,33,83] identified through the data base searches and guided by authors’ knowledge and experience. The use of comprehensive original data sources, in-depth analysis, or their association with respected national bodies with extensive experience in Aboriginal and mainstream health service partnerships (Reconciliation Australia, Secretariat of National Aboriginal and Islander Child Care) were important considerations.

Taylor and Thompson identified 16 key learnings from 24 published reports of AMP on improving AMP in health services [5]. Reconciliation Australia asserted ten ingredients for successful Aboriginal and Torres Strait Islander policies and programs outlined in past reports, studies and research papers [83]. Burton outlined eight core principles underpinning genuine and successful partnerships between Aboriginal controlled community organisations and mainstream service providers from a case study review [33]. Elements in all three sources had some similarities and were useful in providing the lens through which elements in the partnership tools are assessed.

### Partnership tool reviews

Two partnership assessment tool summaries [38,59]; three reviews of partnership assessment tools [61-63] and four papers documenting experiences of implementing partnership analysis tools [57,60,64,84] were downloaded to identify tools relevant to consider for AMP assessment. One comparative analysis of four selected partnership analysis tools was excluded due to a substantial gap in methodology documented [63]. At the time this paper was revised, Markwell’s *Partnership Working: A Consumer Guide to Resources* [38] was no longer accessible through the internet and therefore is not included in Table 2 which summarises the author(s), year of publication, aim of the review, methods of the review, study location and intended audience of the seven partnership tool reviews. None of the identified reviews or discussions made any specific reference to AMP or working in cross-cultural partnerships. The following sections outline relevant learnings from these papers regarding using tools, which inform the subsequent analysis of the identified tools using the AMP principles.

**Table 2:** **Partnership tool review articles downloaded for detailed study**

**Author**	**Year**	**Aim of the review**	**Methods**	**Study location**	**Audience**
Joss N & Keleher H [62]	2011	Reports analysis of online self-assessment partnership tools which have data-generating capacity to determine what they measure and to understand how effective they can be in evaluating collaborative practice.	Criteria for analysis developed from literature review to assess the value that partnership tools provide and determine whether they are worth the time and effort to administer and to what extent they generate meaningful data for future decision making.	Melbourne, Australia	Health promotion and community sector programs
	**Tools reviewed:** The Partnership Analysis Tool (VicHealth Australia); Partnership Self-Assessment Tool (CACSH); The Partnership Assessment Tool (Office of the Deputy Prime Minister, UK); Partnership Tool (FaHCSIA, Australia).				
	**Other notes:** Tool inclusion criteria: Partnership tools had to be self-administering; and the user should incur no cost. Exclusion criteria: Tools that did not generate evaluation data; tools which incurred a download cost to the users; and tools which provided partnership management templates.				
Horton D, Prain G & Thiele G [61]	2009	To explore the current state of knowledge of the actual and potential roles of partnership in international agricultural research for development.	Review of research studies, professional evaluation literature, practitioner-oriented reviews, guidelines, and assessment tools, CGIAR reviews, evaluations and policy documents related to partnership.	Peru	Consultative Group on International Agriculture Research
	**Tools reviewed:** 13 partnership tools reviewed including VicHealth Tool, Nuffield and Markwell.				
	**Other notes:** provides comprehensive summaries of a wide range of partnership tools. Assess partnership literature in general without critique on the partnership tools.				
Granner ML & Sharpe PA [59]	2004	To identify published measurement tools for assessing coalition or partnership functioning, and to report the available evidence for validity and reliability of each.	Review of literature conducted through web-based databases. Internet search through Google search engine to identify tools and reports. Included measures that provide at least a conceptual definition of the construct measured.	Columbia, USA	Health Education Research
	**Tools reviewed:** 146 measurement scales/indexes were identified from six tools (Assessing your collaboration: a self-evaluation tool by Borden and Perkins; The Plan Quality Index by Butterfoss, Goodman & Wandersman; Evaluation rubric from Center for Prevention Research and development; Community coalitions: a self-assessment tool by Goldstein; Empowerment praxis in community coalitions by McMillan etc; Coalition self-evaluation instrument by National Network for Health; Evaluating Collaboratives: research and potential by Taylor-Powell et al.				
	**Other notes:** partnership tools included were dated between 1995 to 2001				
Ball R, Forbes T, Parris M & Forsyth L [57]	2010	To apply developed methodology to evaluate both the ‘process’ and ‘outcome’ aspects of three Community Health Partnerships in Central Scotland.	Development of a methodology based on Hardy and Hudson’s Partnership Assessment Tool with adapted structure to incorporate the views of stakeholders. A modified tool was developed to evaluate outcomes incorporating interview components and objectives of particular importance to the Scottish Executive.	Central Scotland	Community Health Partnership
	**Tool reviewed:** Hardy B, Hudson R, and Waddington E (2003) Assessing Strategic Partnerships – The Partnership Assessment Tool. Strategic Partnership Task Force, Office of the Deputy Prime Minister.				
	**Other notes:** Reporting positive experience of applying an adapted partnership tool.				
Sunderland N, Domalewski D, Kendall E & Armstrong K [84]	2009	Focuses on partnership manager’s observation on the use of a partnership self-evaluation tool in local health partnerships in Australia.	A mix of open-ended questions and 7-point rating scales to gather data on partnership manager’s experience in using an adapted partnership tool. Content domains include uptake of partnership tool, uptake of the partnership tool, utility of partnership tool, validity of the partnership tool and future use of the partnership tool.	Queensland Australia	Australian Local Health Partnerships
	**Tool reviewed**: a tool adapted from the VicHealth Partnership Analysis Tool developed by a private consultant.				
	**Other notes:** Empirical study on partnership manager’s experience of using a partnership analysis tool.				
Serafin R, Stibbe D, Bustamante C & Schramm C [64]	2008	To assess the ‘how and what’ of what concerns partnership practitioners in evaluating the cross-sector partnerships in which they are involved. The motivation was to identify the ingredients of a successful partnership evaluation and to identify priorities for further research and development of tools for evaluating cross-sector partnerships.	A combination of desk research, literature review, questionnaire surveys and face to face interviews.	Longdon, UK	The Partnership Initiative (TPI)
	**Tools reviewed:** This paper contains a section on selection of tools without reviewing any particular tool.				
	**Other notes:** An assertion to justify priority be given for research and development to develop more effective tools, methods, frameworks and approaches for evaluating the totality of performance, benefit and impact of cross-sector partnerships.				
Halliday J, Asthana SNM, & Richardson S [60]	2004	To explore the contribution of formal tools to the understanding of partnership drawing on the experience of applying an adapted tool to two Health Action Zone evaluations.	Documenting experience.	United Kingdom	Area-based initiatives such as Health Action Zones.
	**Tool reviewed**: a tool adapted from the Nuffield Partnership Assessment Tool and the Verona Benchmark.				
	**Other notes:** Discussion on the experience of applying an adapted tool.				

#### Using partnership tools

Typically partnership evaluation frameworks and tools are selected or compiled by the agency, partner or evaluation funder [64] in order to measure the effectiveness of collaborative endeavours and to demonstrate to funding bodies that the partnership has been worthwhile [62]. In keeping with partnership development aims, self-assessment tools are designed to generate discussion among partners although the literature is divided regarding the value of partnership tools. The strengths and weaknesses/limitations in partnership tools identified in the reviewed articles are summarised in Table 3.

**Table 3:** **Strengths and weakness of the use of standardised partnership assessment tools**

**Strengths**	**Weaknesses/limitations**
• Useful in providing ‘snapshots’ on the strengths and weaknesses of partnership practice [62]	• *Provide little in-depth contextual data to assist the reflection on and evaluation of partnership outcomes* [62].
• Provide easily accessible, simple and cost-effective means to measure the basic characteristics of a partnership’s work and the collaborative progress during the lifetime of the partnership [62]	• *Generally inadequately capture the complex, dynamic and context specific nature of partnership working.* Halliday et al. believe that formal tools are open to misinterpretation if used as a ‘stand-alone device’ [60]
• Data obtained can provide a developmental framework for establishing an effective partnership and can be used in all transitional stages of partnership development, including formation [84]	• Overreliance on standardized guidelines and analysis tools may not only deny the complexity and idiosyncrasy of collaborative situations, but risk surfacing the tension and exploring the pluses and minuses of alternative ways of addressing issues [85]
• Partnership tools can help build knowledge and capacity in local communities that endures beyond the periods of funded program implementation, thereby enhancing the benefits gained from local community partnerships [84]	• *Application of partnership tools can be time consuming*
• A structured tool can help to discriminate between performance of different partnerships and also between different aspects of partnership working [57].	• The need to demonstrate ‘value for money’ and tangible outcomes *for partnerships funded by short term government initiatives* can result in the use of an ‘auditing tool’ to show success rather than supporting ongoing development through the exposure and discussion of partnership strengths and weaknesses [84]

#### Suggestions for using tools

The experience of applying a structured tool in partnership evaluation has highlighted conditions to be met in order for a useful application of assessment tools.

The first and most important condition to be met prior to applying a partnership tool is having the understanding of organizational settings and the operational environment alongside any agreed measurement of partnership effectiveness [60].

Secondly, the funding or governing authorities requesting a tool supported partnership evaluation process need to ensure that all partners understand the value of ongoing self-evaluation or reflection for partnership development, and that this should ideally take place in the early formation stage [84].

Thirdly, appropriate time and support should be given to master the technical aspects of using self-assessment tools [84]. It is important to consider whether the chosen method offers a means of analysis for the partnership as an entity as well as meeting the needs of individual partners [64].

Application of partnership tools can be time consuming and require substantial commitment; otherwise it is unlikely to foster learning and development [60]. Partners need to be prepared to invest the necessary resources in broad-based evaluation activities, including predetermining the components of partnership to be measured, the time and effort a partnership is willing to invest in the evaluation process, and the most appropriate way to evaluate the partnership [62].

Finally, as suggested by Granner and Sharpe, valid and reliable tools could be applied across multiple partnerships in the same community context in order to achieve a better understanding of the associated factors which influence partnership functioning and community health impacts and outcomes [59].

#### Adapting partnership tools

A cross-cutting theme from discourses on the role of an assessment tool in partnership evaluation has been the importance of reflecting the context in which the partnership operates [59,60,62,64,85]. Similarly, there is considerable skepticism around the ability of existing tools to generate valid and reliable data to reflects changes in program quality, shifts in success factors and the impact of working in partnership on desired outcomes [62,85]. The question of whether or not, and how, to adapt existing tools is examined in many papers.

Some papers document experiences of using adapted partnership tools to meet the needs of the partnerships in question [57,60,84]. These authors generally warn against over-reliance on a structured tool without contextualizing the assessment findings to the circumstances in which the partnerships are operating [62,84,85]. Other practitioners have chosen from available tools as starting points for adapting or creating an evaluation instrument to suit the circumstances [57,60,84].

Joss and Keleher point out that different tools represent variations in focus rather than intrinsic superiority, so evaluators need to be guided by the requirements of the partnership [62]. They suggest that it is better to design a bespoke tool that reflects the organic and context-specific nature of partnerships and to capture the composite and complex partnership characteristics in a valid and reliable manner [62].

The approach in adapting partnership tools is not well documented. Discussion predominantly focuses on whether the difficulty of adaptation is associated with the design of the instrument in the data collection phase, or if more emphasis should be placed on contextualizing findings in the analysis stage. We return to the discussion of contextualizing findings in the Discussion section of this paper.

### Elements of partnership self-assessment tools

Seven tools were identified that met the inclusion criteria. They were subjected to detailed analysis and the aims, audience and basis of tool elements summarised (Table 4), including reporting whether the basis of the elements came from a review of literature on partnership success factors and/or on practitioner experiences.

**Table 4:** **Partnership tools included for detailed analysis**

**Author**	**Year**	**Name of the tool**	**Aim of the tool**	**Basis of tool elements**	**Audience**	**Location: publisher**
Spink and Merrill-Sands [68]	1999	Successful collaborative partnership: Key elements and a self-assessment inventory	Intended for use either at the start-up phase or later on to reflect on strengths and priorities for improvement.	Literature review and practitioner experience.	CGIAR Centers and their partners	Consultative Group on International Agriculture Research
Mattessich PW, Murray-Close M, Monsey BR & Wilder Research Centre [79]	2001	Wilder Collaborative Inventory (found in *Collaboration: What Makes it Work, 2nd Ed*)	Provide a practical tool that bridges the gap between research and practice.	Review of research literature on factors that influence the success of collaboration.	Groups working on collaborative projects	Minnesota, USA: Fieldstone Alliance
Center for the Advancement of Collaborative Strategies in Health [74]	2002	Partnership Self-Assessment Tool (also known as The New York Partnership Self-Assessment Tool)	To assess how the collaborative process is working and identify focus areas to make the collaborative process work better.	Based on a 2001 national study on partnership synergy involving 63 US partnerships (815 partnership participants).	Broad array of partnerships focusing on any kind of goals	New York, USA: CACSH
Markwell S, Watson J, Speller V, Platt S & Younger T [78]	2003	The Working Partnership Book 1–3 (previously and still common known as the Verona Benchmark)	To self-assess levels of performance in leadership, organization, strategy, learning, resources and programs.	Based on evidence, theory and practice in the areas of business performance assessment, community involvement and partnership dynamics.	UK health sector, inter-government department initiatives.	Yorkshire, UK: Health Development Agency
Hardy B, Hudson B & Waddington E [77]	2003	Assessing Strategic Partnership: The Partnership Assessment Tool (based on the Nuffield Partnership Assessment Tool)	Provide a simple, quick and cost-effective way to assess the effectiveness of partnership working, identify problem areas to inform remedial action and guide resource allocations.	Previous Nuffield Institute work with health and social care partnerships.	Local government authorities	London, UK: Office of the Deputy Prime Minister
VicHealth [82]	2011	The Partnership Analysis Tool	To assist organisations to develop a clearer understanding of the purposes of collaboration, to reflect on the partnership they have established, and to focus on ways to strengthen new and existing partnerships by engaging in a discussion about issues and ways forward.	Based on evaluation initiatives undertaken to promote mental health and wellbeing in Victoria.	Health promotion initiatives	Victoria, Australia: VicHealth
Tennyson, R [81]	2011	The Partnering Initiative’s Partnering Tool Book (4th ed)	To help design, develop and manage cross-sector collaboration in a systematic way in order to achieve effectiveness and sustainability.	Builds on the experience of practitioners and offer an overview of essential elements of effective partnering.	General audience using cross-sector collaboration and partnership to achieve development goals.	The Partnering Initiative (International Business Leaders Forum)

In total, 190 process, relational and outcome elements were identified from the partnership tools, 100 of which were relational elements, 80 were process elements and only 10 were associated with partnership outcomes (data not shown). The outcome elements were not included for the purpose of this analysis.

The process and relational elements of the reviewed tools were mapped against the process and relational elements of partnership functioning, identified through the thematic analysis of the three sets of AMP principles. The identified process elements (themes) are presented together with their respective sources and principle states in the first three columns of Table 5, while those associated with relational elements are presented in the first three columns of Table 6.

**Table 5:** **Key process themes found in the AMP principles**

**Article**	**AMP process principles/items**	**Themes**	**Elements found in partnership tools**
TT	Position staff at partner organisation (staff exchanges)	Staff exchange	No
RA	Co-operative, cross sector approaches which reduce the burden of duplication and red-tape on community organisations.	Cross sector approaches	No
TT	Develop linkage processes, including formal documentation of partnership service structure; clarification of roles and clear lines of who troubleshoots	Partnership structure	Yes
TT	Ensure partnership is built on realistic resource capacity to support development of partnership and execution	Development and implementation resource	Yes
RA	Real investment of dollars and people based on need and ongoing support for programs that work.	Financial and human resource	Yes
B	Aim to improve long-term well-being outcomes for Aboriginal and Torres Strait Islander children, families and communities.	Time Resource (Long term)	Yes
RA	Programs and policy approaches that are geared towards long-term achievements.		Yes
TT	Be consistent with meetings; use innovative communication technologies where necessary to maintain contact	Regular meetings/contacts	Yes
TT	Ensure meetings are held regularly and staff have opportunity to interact and build relationships.		Yes
TT	Give the partnership service an Aboriginal name and ensure there are suitable promotion/materials	Aboriginal Name/Suitable promotion	No
B	A commitment to redressing structures, relationships and outcomes that is unequal and/or discriminatory.	Reflection	Yes
B	Valuing process elements as integral to support and enable partnership.	Valuing process	Yes
RA	Regular and independent public evaluation of programs and policies to make sure we learn from mistakes and successes.	Monitoring and evaluation	Yes
TT	Set targets, develop reliable data collection to simple monitoring and outcome indicators	Monitoring and evaluation	Yes
B	Aim to improve long-term well-being outcomes for Aboriginal and Torres Strait Islander children, families and communities.	wellbeing outcomes	No

TT: Taylor and Thompson [5].

RA: Reconciliation Australia [83].

B: Burton [33].

**Table 6:** **Key relational themes found in the AMP principles**

**Article**	**AMP relational principles/item**	**Themes**	**Elements found in partnership tools**
B	Respect for Aboriginal and Torres Strait Islander cultural knowledge, history, lived experience and connection to community and country.	Respect for Aboriginal culture	No
TT	Ensure non-Aboriginal staff have cultural awareness training and Aboriginal staff have opportunities for professional development.	Cultural exchange	No
TT	Honour Aboriginal ways of building relationships and allowing development of trust over time and mainstream health services	Aboriginal Ways	No
TT	Ensure partnership services are developed in response to needs articulated by the Aboriginal community between Aboriginal.	Responding to community needs	Yes
B	Openness to working differently with Aboriginal and Torres Strait Islander peoples, recognising that the mainstream approaches are frequently not the most appropriate or effective.		Yes
RA	Local and region specific programs that are tailored to the needs of particular communities rather than “one size fits all” approaches.		Yes
TT	Ensure the project that is visible to local community and get them engaged.	Community engagement	Yes
B	Commitment to self-determination for Aboriginal and Torres Strait Islander peoples.	Self-determination	Yes
RA	Genuine engagement with communities in talking about, developing and implementing policies.		Yes
RA	Long-term investment in strengthening communities at a local level to decide and manage their own lives.	Strengthen communities	Yes
B	Commitment to developing long-term sustainable relationships based on trust.	Long term	Yes
RA	Long-term investment in strengthening communities at a local level to decide and manage their own lives.	Long term	Yes
RA	Active and well-supported Aboriginal and Torres Strait Islander led decision-making in program-design.	Resourcing	No
RA	Investment in and support for local Aboriginal and Torres Strait Islander leadership.	Local Leadership	No
TT	Dedicate time for a development period to build mutually respectful relationships.	Mutually respectful relationship	Yes
TT	Ensure there is equal participation in planning and power sharing.	Equal participation	Yes
TT	Need for motivated individuals (partnership champions), commitment of senior staff, leadership and vision.	Leadership	Yes
RA	Grass-roots, bottom-up approaches that knit together local knowledge within a national framework.	Bottom-up	Yes
B	Shared responsibility and accountability for shared objectives and activities.	Shared responsibilities	Yes
TT	Ensure meetings are held regularly and staff have opportunity to interact and build relationships.	Opportunity to interact and build relationships	Yes
TT	Use a facilitator to openly negotiate historical baggage and different approaches to health/ culture. Have a commitment to work through issues using problem solving processes.	Open Communication	No
TT	Use innovative power sharing methods, such as changes in chairing of meetings, place of meetings, etc.	Power sharing	Yes

TT: Taylor and Thompson [5]; RA: Reconciliation Australia [83]; B: Burton [33].

The elements and gaps in mapping to AMP principles are shown in the fourth column of Tables 5 and 6 for process and relational factors respectively.

#### Process elements in partnership assessment tools mapped to AMP principles

Nine of the 15 AMP principles associated with partnership processes were reflected in elements of the reviewed partnership assessment tools. Table 7 provides a summary of the process elements found in the partnership self-assessment tools corresponding with relevant AMP principles.

**Table 7:** **AMP principles and corresponding process elements in existing partnership assessment tools**

**Aboriginal-mainstream partnership principles**	**Article**	**Themes**	**Summary of partnership self-assessment process elements**	**Tools**
Develop linkage processes, including formal documentation of partnership service structure; clarification of roles and clear lines of who troubleshoots.	TT	Partnership structure	Formal and informal communication links; sharing, accessibility and management of data, information and knowledge; open, simple and frequent communication	CGIAR, Wilder, New York PSAT, VicHealth, Markwell(Verona), Tennyson
			Features of good partnership: clarity of roles, responsibilities, procedures, expectations, attention to process.	CGIAR, Markwell (Verona), VicHealth
Ensure partnership is built on realistic resource capacity to support development of partnership and execution.	TT	Development and implementation resources	Relevant skills and expertise, agree on policy and the level of funds, human and material resources required.	Wilder, Nuffield, New York PSAT, VicHealth, Markwell(Verona), Tennyson
Real investment of dollars and people based on need and ongoing support for programs that work.	RA	Financial and human resource		
Be consistent with meetings; use innovative communication technologies where necessary to maintain contact	TT	Regular meetings/contacts	Consistency of Commitment	Nuffield
Ensure meetings are held regularly and staff have opportunity to interact and build relationships.	TT		Flexibility and adaptability - flexible enough to allow participation of all players; adjust time, place and organisation of partnership meetings to minimize barriers to participation.	Wilder, New York PSAT, VicHealth, Markwell (Verona)
A commitment to redressing structures, relationships and outcomes that is unequal and/or discriminatory.	B	Reflection	Commitment to reconsider and modify aim, objective, policy and strategies based on review findings.	Nuffield, Markwell (Verona)
Valuing process elements as integral to support and enable partnership.	B	Valuing process	Prime focus on process, outcome and innovation	Nuffield, VicHealth
Regular and independent public evaluation of programs and policies to make sure we learn from mistakes and successes.	RA	Monitoring and evaluation	Identify success factor and barriers to partnership work including past successes; better utilisation of available skills and expertise; information provision including orientation and contextual materials to support timely decision; skills development including participatory skills, partnership monitoring and reviewing skills.	Nuffield, New York PSAT, VicHealth, Markwell (Verona)
Set targets, develop reliable data collection to simple monitoring and outcome indicators	TT		Define clear service outcomes; Shared vision and mission (goals, aims, objectives): clearly communicated to the community, compelling, concrete, attainable; agreed principles and approaches in addressing the defined problems.	CGIAR, Wilder, Nuffield, NYPSAT, VicHealth

TT: Taylor and Thompson [5].

RA: Reconciliation Australia [83].

B: Burton [33].

In terms of process assessments in AMP, the elements in the reviewed tools concerning common aims, partnership and membership structure, communication, valuing process, outcome and innovation, flexibility and adaptability to ensure involvement of all partners and the reflective elements of monitoring and evaluation are all applicable in the AMP context.

The key deficiency in process elements found in the tools reviewed with reference to the AMP principles related to the timeframes and level of resourcing required to achieve determined outcomes. The three sources of the AMP principles emphasise realistic investment [5,83] for long-term achievements [83] and long-term wellbeing outcomes [33]. Whilst the tools reviewed also highlighted the importance of realistic and adequate resourcing [74,77,78,82], the focus has been on the agreed level of resourcing, outcome and achievements by all partners. Thus, the timeframe in which resources should be allocated appears to be a point of distinction in Australian AMP circumstances.

#### Relational elements in partnership assessment tools mapped to AMP principles

Sixteen of the 22 AMP principles associated with relational elements were reflected in the partnership assessment tools included in this review (Table 8).

**Table 8:** **AMP principles and corresponding relational elements in existing partnership assessment tools**

**Aboriginal-mainstream partnership principles**	**Article**	**Themes**	**Summary of partnership self-assessment relational elements**	**Tools**
Ensure partnership services are developed in response to needs articulated by the Aboriginal community	TT	Responding to community needs	Connections to community: prioritise local concerns, respond to needs and problems of the community.	New York PSAT; Markwell (Verona)
Grass-roots, bottom-up approaches that knit together local knowledge within a national framework.	RA			
Openness to working differently with Aboriginal and Torres Strait Islander peoples, recognising that the mainstream approaches are frequently not the most appropriate or effective.	B		Implement strategies most likely to work in the community.	New York PSAT
Local and region specific programs that are tailored to the needs of particular communities rather than “one size fits all” approaches.	RA			
Commitment to self-determination for Aboriginal and Torres Strait Islander peoples.	B	Self-determination	Community influence: community representatives can influence partnership decisions;	Markwell (Verona)
Long-term investment in strengthening communities at a local level to decide and manage their own lives	RA		Include the views and priorities of people affected by the partnership’s work/use participatory methods to stimulate active community engagement in planning.	New York PSAT
Genuine engagement with communities in talking about, developing and implementing policies.	RA		support from potential blockers.	New York PSAT
Ensure the project that is visible to local community and get them engaged	TT	Community engagement	Heighten public profile and added prestige for the collaborative as well as the individuals.	New York PSAT; VicHealth
Dedicate time for a development period to build mutually respectful relationships	TT	Mutually respectful relationship	Mutual respect and understanding: inclusiveness, openness, encourage innovation to develop roles in local communities.	Wilder; Markwell (Verona)
Commitment to developing long-term sustainable relationships based on trust	B	Sustainable relationships	Develop valuable relationship: communicate partnership vision; develop common language, inclusive decision making.	New York PSAT; VicHealth; Tennyson
Ensure there is equal participation in planning and power sharing	TT	Equal participation	Involvement in planning and setting priorities; fairness in conduct of partnership.	Nuffield; VicHealth
			Difference in opinion, individual interest freely expressed; fairness in distribution of benefits.	Nuffield; New York PSAT; VicHealth; Markwell (Verona)
Need for motivated individuals (partnership champions), commitment of senior staff, leadership and vision	TT	Leadership	Have roles that cross the traditional boundaries.	VicHealth
Investment in and support for local Aboriginal and Torres Strait Islander leadership.	RA		Inspire, motivate and empower people to be involved.	New York PSAT
Use innovative power sharing methods, such as changes in chairing of meetings, place of meetings, etc.	TT	Innovation	Identify new and creative ways to solve problems	New York PSAT
Shared responsibility and accountability for shared objectives and activities.	B	Shared responsibilities	Mutual accountability: members share a stake in both process and outcome; clear lines of accountability for performance.	CGIAR; Wilder; Nuffield; Tennyson

TT: Taylor and Thompson [5].

RA: Reconciliation Australia [83].

B: Burton [33].

A good alignment was found between elements of mutual respect/trust, equal participation/democracy and equity, innovation/creativity in power sharing and problem solving, and sharing of accountability and responsibility. The cross-cutting theme in the leadership elements involves a need to nurture motivated individuals not only to work in partnerships but also to have the skills and preparedness to cross traditional boundaries, a role referred to as the ‘boundary spanner’ in a number of partnership discourses [58,86,87]. Elements associated with equal participation found in the Nuffield, VicHealth, New York PSAT and Verona benchmark tools are all potentially appropriate to assess the AMP principle of equal participation in planning and power sharing found in Taylor and Thompson [5]. Elements of shared responsibility found in the Wilder Collaborative Inventory [79] are also potentially appropriate in this context.

Mapping exercises contained in two of the reviewed tools appear helpful in unpacking the complex relationships in which some partnerships operate. The VicHealth Tool is a simple but useful activity for mapping partnership, assessing the nature of relationships between participating partners, and differentiating between networking, coordinating, cooperating and collaborating relationships [82]. Tennyson’s partnering tool book proposes mapping stakeholders according to the level of interest and degree of influence of each partner [81].

There are also items extending the analysis beyond internal partnership relationships to include relational aspects of the partnership with the community and broader society. Community understanding, partners’ perceptions that the partnership’s operation and achievements are meeting the needs of the community and stakeholders, and that there is adequate understanding of these benefits among community members are included. The impact of the historical, social and political environment in which the partnership operates is also subject to assessment, including issues such as the distribution of resources, class and community, gender and race. Connections between the partnership and political decision makers and external organisations are also viewed as conducive to successful collaboration [74,78].

Elements associated with self-determination found in the Verona benchmark [78] and New York PSAT [74] are comprehensive and potentially useful in the Australian AMP context. Similarly, elements associated with community engagement found in the New York PSAT and VicHealth tools have potential to support and analyse aspects of sustainable relationships (Table 8).

Elements associated with the AMP principles of respecting the needs of the Australian Aboriginal and Torres Strait Islander communities were less neatly aligned. Elements associated with responsiveness to community needs (Table 8) should reflect being responsive to the needs articulated by the Aboriginal community [5], working with Aboriginal people to knit together local knowledge within a national framework [83], and openness to approaches that are different from the mainstream conventions [33,83]. While these principles found their counterparts in elements of the New York PSAT and the Verona benchmarking tools, they are generally poorly captured.

One of the recommendations made to improve AMP is related to visibility of partnership projects in order to ensure engagement by the members of the local communities [5]. Whilst the New York PSAT and the VicHealth tools have corresponding elements associated with raising public profile and prestige of the partnership, they are referring to ‘public profile’ in different contexts. Whether or not the purpose of such an element is to encourage participation by potential partners is not apparent from the literature reviewed.

Cultural components in the AMP principles include elements of respecting Aboriginal cultural knowledge, lived experience and honouring the ‘Aboriginal ways’ and assessment of these was not available in any of the tools reviewed. Furthermore, openly negotiating what Taylor and Thompson refer to as “historical baggage” [5] is another element specific to the Australian AMP context not captured in the reviewed tools.

### Alignment between partnership tools and AMP principles in the Australian context

Table 9 summarises the number of corresponding AMP principles the process and relational elements contained in each of the tools reviewed.

**Table 9:** **Summary of process and relational principles associated with elements in partnership tools**

**Name of partnership tools**	**Number of Aboriginal-mainstream partnership principles associated with the tool elements**		**Total**
	**Process**	**Relational**	**Process plus relational**
Successful collaborative partnership: Key elements and a self-assessment inventory (The CGIAR Tool) [68]	3	1	4
Wilder Collaborative Inventory (found in *Collaboration: What Makes it Work, 2nd Ed*) [79]	5	2	7
Partnership Self-Assessment Tool (The New York Partnership Self-Assessment Tool) [74]	5	6	11
The Working Partnership Book 1–3 (The Verona Benchmark tool) [78]	6	3	9
Assessing Strategic Partnership: The Partnership Assessment Tool. (The Nuffield Partnership Assessment Tool) [77]	7	3	10
The VicHealth Partnership Analysis Tool [82]	7	5	12
The Partnering Initiative’s Partnering Tool Book (4th Edn) by Tennyson [81]	2	2	4

Five out of seven tools have potential to assess the process aspects of Aboriginal-mainstream partnerships. Hardy et al’s Nuffield Partnership Assessment Tool [77] and the VicHealth Partnership Analysis Tool [82] contain elements corresponding to seven of the principles. Markwell et al’s Working Partnership Book [78], previously known as the Verona Benchmark, contain elements corresponding to six principles, while the Wilder Collaborative Inventory developed by Mattessich et al. [79] and the New York Partnership Self-Assessment Tool [74] contain elements corresponding to five of the AMP ‘process’ principles.

Two of the seven tools have greater potential to assess the relational aspects of AMP principles. The New York Partnership Self-Assessment Tool [74] contains elements corresponding to six AMP ‘relational’ principles, while the VicHealth Partnership Analysis Tool [79] contains elements corresponding to five principles. In addition, the Wilder Collaborative Inventory contains elements corresponding to mutual respect and accountability which distinguished it from the New York PSAT and VicHealth tools which did not assess these elements.

Overall assessment indicated that the New York PSAT and the VicHealth Partnership Analysis tools reflect the greatest numbers of AMP principles followed by the Nuffield Partnership Assessment tool. The New York PSAT has the strongest alignment with the relational elements of the AMP principles adopted in this paper while VicHealth and Nuffield tools showed greatest alignment with the process elements in the chosen principles.

## Discussion

The focus on relational and process elements in the partnership tools reviewed is consistent with the focus of Australian AMP Principles by reconciliation advocates. The potential of a large number of elements contained in available partnership self-assessment tools has been affirmed. However, the historical context, lived experience, cultural context and approaches of Australian Aboriginal people represent key deficiencies in the tools reviewed.

A history of oppression is not a distant memory to the Australian Aboriginal population remaining part of their lived experience. It is important to recognise and respect the world views of Aboriginal and Torres Strait Islander people [88] when interpreting the process, relational and outcome data collected. Relevant to the current Aboriginal and mainstream partnership (AMP) discourse is Johnstone’s argument that advocates for health researchers to engage in the distinctive political process of ‘recognition’ and ‘reconciliation’,

‘*If the future of Indigenous health research is to be strengthened, researchers must confront rather than deny the past………researchers need to engage in the political process of reconciliation….As a matter of human decency, researchers (even though they may have had nothing to do with the past abuses), should express profound sorrow for those ‘dark bits’* [89]

Further, within some AMP it has been agreed that the partnership not be “equal” in the sense that the Aboriginal worldview and agency will be privileged, with mainstream evidence and energy used in a complementary way, for what it can add [90]. This may be a necessary approach in a period where power and privilege are being redressed and balanced, but is likely to require a substantially different assessment tool to those reviewed. In this context, relational elements with particular reference to the sociocultural and political context of partnership working will be particularly important [91] in addressing data collection and analysis issues. This in turn supports the argument for the development of an appropriate assessment instrument.

### Bespoke tool or adapting a tool

Rather than constructing a tool by compiling elements from various tools, the analysis in this review has demonstrated the potential to use either the VicHealth Partnership Analysis Tool or the New York PSAT as starting points. Where elements of mutually respectful relationship and sharing of responsibilities are weaker in these tools, associated elements can be borrowed from other tools as summarised in Table 8 to ensure comprehensive coverage of elements of working partnerships. The relevant parts of the New York PSAT together with Verona Benchmark tool also offer a good inventory for community self-determination which is emphasised in the Burton, Reconciliation Australia and Taylor and Thompson principles [5,33,83].

Developing and maintaining a successful AMP requires mutual learning processes and the comparative analysis in this review identified opportunities for mainstream partnership assessment to incorporate learnings from Aboriginal-mainstream partnerships. Whilst the New York PSAT and the Verona benchmark contain corresponding elements associated with responsiveness to community needs, elements in the AMP principles suggest there are opportunities to enrich the community centred culture elements in these tools in order to support community-based programs (Table 8).

Given the global reality of the inadequate life-span of funded partnership programs, existing partnership tools have placed emphasis on ‘agreed’ levels of resourcing and realistic outcomes for the partnerships to achieve. This, however, does not reflect the AMP principles. In the Australian context, policy makers and practitioners have recognised the significance of sustainable trusting relationships and the need to set long term goals, aim for long term achievements and long term investments in partnerships initiatives [33,83]. This presents an opportunity to integrate structured assessment to monitor changes in partnership process and relations to support longer term changes in the desired outcomes to be achieved.

### Contextualising partnership assessment tools findings

The key challenge faced in applying structured tools to assess partnership working has been on contextualisation of findings. An unresolved question is whether it is possible to capture the complex dynamics using a structured partnership analysis tool even if using a bespoke tool designed for the specific purpose of capturing aspects of AMP. The answer is uncertain; however, elucidating the historical, sociocultural and political background of Australian AMP when interpreting data collected using structured tools is essential to ensure that findings are as close to reality as possible. If the underpinning rationale for the drive to work in partnerships in this special context is to redress and balance power and privileges between the Aboriginal and mainstream partners, then discussions of equity and equality must be included when analysing findings from partnership assessments. This also has implications for the dissemination of findings.

The impact of current policy and sociocultural environments, and the power dynamics which operate in Aboriginal and mainstream settings are of crucial importance when building and maintaining AMP [37]. That is, the political, economic and social disadvantage of Aboriginal and Torres Strait Islander people in Australia is especially important to consider when assessing the health of the partnerships. Therefore, tools to evaluate partnerships need to include culturally appropriate and community relevant outcomes. If the criteria related to partnership success are not culturally appropriate for an AMP or do not reflect the social and political context, then the quality and appropriateness of the data collected must be questioned.

Regardless of the type of tool used, the importance of complementing the data collected with a broader examination of relevant public policy, service delivery, and community outcomes must be acknowledged. All assessment should be placed in a broader social ecological context which recognises that various levels of the political environment can impact on the effectiveness of self-determination and genuine AMP. Instead of focusing only on equality in participation and accountability, similarities as well as differences in basic conditions affecting access to information, knowledge, resources and services must be made transparent in order to achieve equity.

An emerging example from the above analysis (Table 8) may allow us to infer on the elements of nurturing leadership quality in this special context. One of the AMP principles involves investment in and support for local Aboriginal and Torres Strait Islander leadership [83] when its mainstream counterpart highlights the importance of ‘initiatives to inspire, motivate and empower people to be involved’ as the key to nurturing leadership [74]. This could mean that when mainstream organisations partner with Aboriginal communities, greater emphasis should be placed on understanding the culture of Aboriginal and Torres Strait Islander leadership while inspiring, motivating and empowering mainstream counterparts to work collaboratively in the AMP context.

### Data collection considerations

The role of the facilitator in implementing a partnership evaluation tool should not be underestimated. Any partnership evaluation is reliant on the sensitivity and awareness of the facilitator and the data analysis process to surface the true underlying issues. For example, people who are reluctant to speak in a public forum may need to be encouraged to participate, or have a more low key opportunity to contribute created for them.

There are barriers to using a partnership tool and participants require preparation to use them in a way that can contribute to high quality sustainable local partnerships [84]. Flexibility must be allowed in terms of the stages in which the tool is applied and how to ensure maximum benefits to strengthen the partnership.

In the initial phases of introducing a partnership tool to an AMP, effort to promote the idea of testing a structured tool to help guide partnership analysis is needed. This means allowing time to assess whether the right questions have been asked to explore partners’ experiences in the context of a wider partnership networks that include local advocacy groups and state, and even federal level government agencies. In the context of Aboriginal-mainstream partnership, it is imperative that the entire evaluation process, starting from data collection, is not separated from the historical, social and political context in which the partnership operates [37].

Long term relationships and trust are especially important in the development and evolution of Aboriginal-mainstream partnerships [5,92]. Even if there is an arbitrary starting point assigned, a genuine partnership rarely has a neat starting point. Partnerships often start with a small number of individuals, the boundary spanners, and in some instances, a desirable partnership outcome may be the increased number of partners willing to participate in the assessment process. This questions the feasibility of a ‘before’ and ‘after’ comparison when that has been deemed necessary. It is more than likely that a tool is most useful to help structure informal conversations to achieve a balance between systematic understanding of partnership dynamics and conversational nature of data collection in this context.

## Conclusion

In conclusion, partnership tools are instruments to help work out where and why the partnership is working or not working. The willingness of partners to engage in any formal self-assessment process in itself suggests the partnership is robust. Tools offer opportunities for providing evidence based support to partnership development. Evaluation of any partnership is really only a means of strengthening the collaboration, and the assessment or analysis process relies upon honesty and openness from the partners and a preparedness to change ways of working if the needs of partners are not being met adequately. It is likely that where these prerequisites are met, the partnership is already on a secure footing, but further improvements may still be possible.

The unique nature of partnerships means an established tool that has been shown efficiency in other contexts (AMP or non-AMP) may not realise the same or may be difficult to implement in some AMP. The multiplicity of tools in existence and the reported uniqueness of each partnership, means the development of a generic partnership analysis tool for AMP may not be a viable option for future effort.

No documented evidence was found in the use of partnership tools in an AMP setting. However, the use of a structured tool, particularly when adapted or used in combination with other data collection techniques to explore the context of program or community development evaluations may add value to partnership assessment. Future research should focus on documenting experience in the application of partnership tools to support Aboriginal-mainstream partnership operations using an appropriate interpretive framework that is cognisant of the factors involved in the process of recognition and reconciliation, including cultural context, self-determination, mutuality and equity.

Haynes and colleagues [93] have argued that reflexivity and dialogical theory are essential theoretically informed ways to work in practice that ensure attention is paid to the nature of partnerships in terms of power, strangeness, borders and intercultural relations [93]. Working in the space of AMP building and improvement, partners, evaluators and researchers face the challenges of getting the balance right between contrasting cultures and customs and the efficacy and efficiency of the process of partnership analysis. The inherent challenges of operating in the intercultural space contribute to the hurdles of applying either established protocols or adapted tools in this highly unfamiliar area in evaluation. As the reason for assessing AMP should be to support long term sustainable relationships based on trust, this strive for balance is an on-going process.

## Abbreviations

ACCHS: Aboriginal Community Controlled Health Service
AMP: Aboriginal-mainstream Partnership
PSAT: Partnership Self-Assessment Tool
VicHealth: The Victorian Health Promotion Foundation
